# Investigation of Virulence and Antibiotic-Resistance of *Bacillus cereus* Isolated from Various Spices

**DOI:** 10.1155/2023/8390778

**Published:** 2023-05-09

**Authors:** Sahar Torki Baghbadorani, Ebrahim Rahimi, Amir Shakerian

**Affiliations:** ^1^Department of Food Hygiene, Faculty of Veterinary Medicine, Shahrekord Branch, Islamic Azad University, Shahrekord, Iran; ^2^Research Center of Nutrition and Organic Products, Shahrekord Branch, Islamic Azad University, Shahrekord, Iran

## Abstract

Spices and herbs are potential vectors for virulent and pathogenic micro-organisms, which cause illness in consumers, contribute to spoilage, and reduce the durability of foodstuffs. The present study aims to provide relevant data about virulence and antibiotic resistance of *Bacillus cereus* isolated from various spices. A total of 200 samples of 8 types of spices (black pepper, chilli, white pepper, cumin, cinnamon, turmeric, curry powder, and sumac) were collected from various markets, retail shops, and sucuk production premises located in the Isfahan province of Iran. Presumptive *B. cereus* strains were obtained using Bacara Agar plates after enrichment in saline peptone water and final colonies were identified using matrix-assisted laser desorption/ionization time-of-flight mass spectrometry. Enterotoxin (HBL) and nonhaemolytic enterotoxin (NHE) production were assessed using the Duopath® Cereus Enterotoxins Test kit. The Kirby–Bauer disc diffusion method was applied as antibiotics susceptibility test. PCR was used to detect Emetic toxin gene (*CES* and *CER*) and enterotoxigenic toxin gene (*cytK, nheA, hblC,* and *entFM*). Results show a significant prevalence of *B. cereus* (42%) in spices. However, the spices meet food safety recommendations (<10^4^ cfu/g). Antibiotics susceptibility test show alarming rate of resistance to beta-lactam antibiotics specially ampicillin (83.33%) and penicillin (82.14%). Concerning the toxin producing capacity more than half of the isolates (51.19%) produce NHE toxin and 27.38% produce HBL toxin. The most abundant gene were *nheA, nheB*, and *nheC* and a combination of 4 genes (*entFM*, *nheA*, *hblC*, and *cytK*) was detected in many isolates. In conclusion, the presence of multidrug resistant *B. cereus* strains carrying diarrhoeal toxin-encoding genes in spices intended for human consumption represents a serious health hazard. These results indicate the need for regular surveillance of the occurrence of *B. cereus* strains in spices and food products in Iran.

## 1. Introduction

*Bacillus cereus* is a member of *B. cereus* group and is a Gram-positive spore-forming bacteria widely found in the natural environment and in foods [1]. This bacterium can survive during the drying and heating processes [2] and its vegetative cells can poison food [3]. Indeed*, Bacillus cereus* is one of the main species contributing to food-borne diseases [4]. *B. cereus* has been ranked among the top ten agents involved in food and water poisoning [5]. Ingestion of *B. cereus* can cause diarrhea or vomiting depending on the toxins produced. The enterotoxins produced by *B. cereus* are heat-labile and cause abdominal pain or diarrhea, while the emetic toxins are heat-stable and cause nausea and vomiting [6]. Of the enterotoxins, Haemolysin BL (HBL) and nonhaemolytic enterotoxin (NHE) are involved in most of the food illness reported. The ability to produce enterotoxins is specific to isolates [7]. Among the variety of foods, contamination of herbs and spices by *B. cereus* are frequently reported [5].

Spices are a widely used condiment in cooking because of their contribution to the taste, smell, color, and preservation of food [3]. They can sometimes be subject to contamination by various micro-organisms due to poor sanitary conditions, environmental factors, and insufficient decontamination [8]. Contamination can occur before, during, and after harvesting, but especially during drying [9]. According to Allende et al., [10] many spice-related food-borne illness outbreaks worldwide have been reported. Apart from these, spices are added to food products. Thus, it is therefore possible that there are more food poisoning cases for which the source could not be determined, or which are not associated with spices [3]. *B. cereus* can survive for a long time in spices due to its ability to withstand an unfavourable environment [2]. Numerous studies have reported the presence of *B. cereus* in spices [7, 9–12].

Spices and herbs are mainly grown and harvested in regions with a tropical climate [6]. Thus, due to the appropriate geographical characteristics, a wide range of spices such as curry, sumac, ginger, and saffron can be grown in the Mediterranean and Middle Eastern regions [13]. Iran is one of the world's leading importers and consumers of spices [14]. Regarding that, data about isolation of *B. cereus* from spices in Iran are limited. Considering that food poisoning by this bacterium is an important public health concern, this study aims to provide relevant data about virulence and antibiotic resistance in *Bacillus cereus* isolated from various spices available in Isfahan, Iran.

## 2. Methods

### 2.1. Sample Collection

The samples were collected from January to December 2021 from different spice outlets in Isfahan, Iran. Spice varieties such as black pepper, chilli, white pepper, cumin, cinnamon, turmeric, curry and sumac were included in the sampling. The number of 25 samples of each type of spice were collected, making a total of 200 samples. The samples were collected under aseptic conditions and sent to the laboratory.

### 2.2. Isolation of *B. cereus*

Isolation of *B. cereus* was performed following method described by [15]. Bacara agar (bioMérieux, France) was used to detect *B. cereus* colonies after diluting each sample with peptone salt water (10^−1^). Incubation condition was 30°C for 48 h. After incubation, presumptive colonies were collected (pink/orange with halos). Identification was performed on each presumptive colony type using matrix-assisted laser desorption/ionization time-of-flight mass spectrometry (MALDI-TOF-MS). Each type of presumptive colony was analysed.

### 2.3. Detection of *B. cereus* Enterotoxin Producers

Enterotoxin production (Haemolytic enterotoxin (HBL) and nonhaemolytic enterotoxin (NHE)) was assessed using the Duopath® Cereus Enterotoxins Test kit (Merck, Belgium) according to the manufacturer's instructions [15].

### 2.4. Antimicrobial Susceptibility Test

The disc diffusion method was applied using Mueller–Hinton Agar (Oxoid, UK) (EUCAST, 2015). The following antibiotic disks were used: ampicillin (AMP, 10 *μ*g), penicillin *G* (10 U), tetracycline (30 *μ*g), erythromycin (15 *μ*g), kanamycin (30 *μ*g), chloramphenicol (30 *μ*g), neomycin (30 *μ*g), oleandomycin (15 *μ*g), cephalothin (30 *μ*g), streptomycin (10 *μ*g), polymyxin B (300 U), and vancomycin (30 *μ*g). *B. cereus* ATCC 10876 was used as control strain.

### 2.5. Detection of Virulence Factors

Isolates identified as *B. cereus* strains were subcultured on Tryptone Soy Agar (TSA) and incubated for 18–24 h at 30°C. DNA extraction kit PrepMan UltraSample Preparation (PrepMan Ultra, Thermo Scientific, Waltham, USA) was used to extract genomic DNA from *B. cereus* isolates following the manufacturer's instructions. A 100 microlitre suspension was prepared with PrepMan Ultra sample preparation reagent and isolated colonies in microcentrifuge tubes. The suspension was vortexed for 10–30 s and the incubation step (10 min at 100°C) and centrifugation (3 min at 14,000 rpm) were applied successively. Total DNA was measured at an optical density of 260/280 nm using a spectrophotometer (Thermo Scientific, Waltham, USA). A concentration of 10 ng/ml of DNA was considered for the PCR step (Biometra, Montgomery, USA). Virulence factors including genes encoding production of emetic toxin (*CES* and *CER*) and enterotoxigenic toxin (*cytK, nheA, hblC,* and *entFM*) were detected. Same primer sequences with product size and PCR conditions were used according to Kim et al. [16]. PCR assay was conducted using a thermal cycler (Mastercycler Gradient S; Eppendorf). The amplification conditions were as follows: initial denaturation at 95°C for 10 min, 35 cycles of 94°C for 1 min, 54°C for 1 min, and 72°C for 1 min, and finally a 5 min extension at 72°C. Negative control was DNase and RNase free water (SigmaAldrich, Munich, Germany). DNA fragments were analysed by electrophoresis using QiAxcel Advanced facility (Qiagen, Hilden, Germany) according to the manufacturer's instructions.

### 2.6. Detection of Component L2 of the Hbl Toxin

The *B. cereus* isolates were incubated on TSA for 18–24 hours at 30°C, and a colony from each sample was then transferred to 10 ml of BHI and incubated for 6–18 hours at 37°C. After incubation, the samples were centrifuged for 20 minutes at 4°C. To determine the production of Hbl, a toxin agglutination test (BCET-RPLA) was conducted following the manufacturer's instructions. This test measures the *B. cereus* L2 component of the Hbl toxin complex. Six half-serial dilutions were prepared for each isolate of the sample, and the results were expressed using indexes ranging from 0 to 128. The results of the BCET-RPLA test were reported as the last dilution of the sample that gave a positive agglutination, with samples reported at 0 dilution being considered negative [6].

## 3. Results

### 3.1. Isolation of *B. cereus* and Bacterial Count

Table 1 shows the results of the presence of *B. cereus* in the 200 spice samples collected. Of the 200 spice samples, 84 samples (42%) were positive for *B. cereus*. A high number of positive samples was observed for black pepper [17] followed by turmeric [16]. The Min log10 CFU/g ± SD^a^ and the Max log10 CFU/g ± SD^a^ were, respectively, ranged from [1.1 ± 0.15; 3.15 ± 0.05] to [3.52 ± 0.11; 4.38 ± 0.15]. Cumin has the highest value of Min log10 CFU/g ± SD^a^ (3.15 ± 0.05) and Max log10 CFU/g ± SD^a^ (4.38 ± 0.15).

### 3.2. Determination of Enterotoxin Producing *B. cereus*

Table 2 presents the results of bacterial count and determination of enterotoxin producing *B. cereus* from spices. According to this table, all *B. cereus* count for cumin is more than 3 log10 CFU/g. The highest *B. cereus* count was noted from two white pepper samples (4.11 and 4.21 log10 CFU/g). More than half of the isolates (51.19%) produce NHE toxin and 27.38% can produce HBL toxin. 20.23% of isolates were found to produce both NHE and HBL toxin. A high number of isolates (73.91%) which produce HBL also produce NHE toxin.

### 3.3. Antibiotic Resistance Pattern of *B. cereus* Strains

Table 3 presents results of the antibiotic resistance pattern of *B. cereus* strains. A high number of isolates exhibited resistance to ampicillin (83.33%) and penicillin G (82.14%). Almost all the isolates were susceptible to polymyxin B, cephalotin, kanamycin, neomycin, streptomycin, oleondamycin, chloramphenicol, imipenem, and nalidixic acid.

Table 4 present the different resistance profile of *B. cereus* isolates. Some isolates from black pepper samples were resistant to 8 different antibiotics and isolates which showed resistance to at least five different antibiotics were found from black pepper [8], chilli [6], curry powder, and white pepper [5].

### 3.4. Detection of Virulence Factors

Table 5 presents the PCR positive samples. More than the half of the samples has either one, two, or three types of NHE. Only 9 samples were positive for FM.

Table 6 presents the results of the detection of some genes related to enterotoxin production. Combination of 4 genes (*entFM*, *nheA*, *hblC*, and *cytK*) was detected in some isolates.

## 4. Discussion

The geographical characteristics contribute largely to the production of a wide range of spices in Iran [13]. This study aims to provide relevant data about virulence and antibiotic resistance in *Bacillus cereus* isolated from various spices available in Isfahan, Iran.

The results for the isolation of *B. cereus* in spices show that of the 200 spice samples, 84 samples (42%) were positive for *B. cereus*. More than 30% percent of recovery rate of *B. cereus* in spices was reported in Turkey [3], USA [7], Latvia [6], and Poland [18]. This commonly reported significant prevalence is supported by the faculty of *B. cereus* to survive in an inappropriate environment such as spices with low water activity and to resist at boiling temperature. Indeed, *B. cereus* spores are extremely resistant to physical and chemical treatments [17]. It can therefore grow rapidly under nonaseptic storage conditions, contaminate food when added to foodstuffs, and cause serious ills [19]. Spices are in powder form, and microbial contamination can occur during handling, processing, transport, and storage [20]. A high number of positive samples was observed for black pepper (18 = 72%) followed by turmeric (16 = 64%). Similar observation was made by Aksu et al. in Istanbul [21]. More positive samples were obtained for cinnamon, thyme, and cumin in another study [3]. Due to their herbaceous nature, all these spices are in regular contact with the soil and therefore can be easily contaminated. These results also highlight the limitations of decontaminating traditional postharvest methods still used in many developing countries [8]. The Min log10 CFU/g ± SD^a^ and the Max log10 CFU/g ± SD^a^ were, respectively, ranged from [1.1 ± 0.15; 3.15 ± 0.05] to [3.52 ± 0.11; 4.38 ± 0.15]. The highest *B. cereus* count was noted from two white pepper samples (4.11 and 4.21 log10 CFU/g). Nanteza et al. also reported almost similar concentration (5.77 log10 cfu/g) of *B. cereus* in white pepper in Indonesia. However, the number of positive white peppers samples is low, 24%, [6] in our study compared to this study, 90% [22]. Several other researchers found high counts of *B. cereus* in white peppers including [23]. Although the highest number of positive spices was found for black peppers, surprisingly the lowest *B. cereus* count (1.1 ± 0.15 log10 CFU/g) were noted for the latter. Similarly, Fogele et al. [6] found the most contaminated spices were black ground peppers. According to Karsha and Lakshmi [24], black pepper inhibits the growth of certain bacteria including *B cereus* thanks to its alkaloid compounds and it can explain the low count observed in this spice. These findings are in line with many studies which also reported low count (<10^4^ cfu/g) of *B. cereus* in the samples analysed [7, 9, 11, 12]. *B cereus* can cause food-borne disease when its concentration in food is of 10^5^ cfu/g or higher [5]. Thus, the foods analysed in this study meet these food safety recommendations.

The results concerning detection of *B. cereus* enterotoxin producer isolated from spices showed that more than half of the isolates (51.19%) produce NHE toxin and 27.38% can produce HBL toxin. These percentages do not correspond to the results of Berthold-Pluta et al. [18] who reported that the percentages of *B. cereus* producing toxins ranged for HBL toxin from 45.9 to 57.9% and for NHE toxin from 61.1 to 81.4% among strains isolated from plant products. This difference can be explained by the number of samples included in our study (200) which is relatively small compared to many other studies. The studies all agree that the percentage of NHE-producing strains is higher than that of HBL producing strains in all sample types [25–27].

Antibiotic resistance pattern results show that a high number of isolates exhibited resistance to ampicillin (83.33%) and penicillin G (82.14%). Similarly, high resistance of *B. cereus* to beta-lactam antibiotics such as penicillin and ampicillin has been reported on several occasions [28]. Indeed, *Bacillus cereus* is well known for its ability to produce *β*-lactamase enzymes [29–31]. Almost all the isolates were susceptible to polymyxin B, cephalotin, kanamycin, neomycin, streptomycin, oleondamycin, chloramphenicol, imipenem, and nalidixic acid. Fiedler et al. [28] also remarked in his study that *B. cereus* strains were susceptible towards antibiotics, including, chloramphenicol and imipenem. Concerning multiresistance pattern profile, some isolates from black pepper samples were resistant to 8 different antibiotics and isolates which showed resistance to at least five different antibiotics were found from black pepper [8], chilli [6], curry powder, and white pepper [5]. This is consistent with Luna et al. [32] and Hwang et al. [33] who also reported multiresistance pattern for some isolates [27]. The presence of antibiotic resistant *B. cereus* especially MDR strains in food commodities indicate the role of food systems as reservoir for resistance genes [34].

The detection of toxin coding genes among *B. cereus* isolated showed that more than the half of the samples has either one, two, or three types of *nhe*. This result is in line with Jovanovic et al. who found high number of *nhe* genes carriers among *B. cereus* isolates [35]. The presence of these genes confirm that they are the most prevalent toxin coding genes among *B. cereus* isolates [6, 33]. The most abundant genes were *nheA, nheB*, and *nheC*. According to Lindbäck et al., [36] *nhe* genes are the most toxic. The virulence pattern of *B. cereus* strains depends on the simultaneous presence and expression degree of the toxin genes rather than the presence of a single gene, each strain has been evaluated individually in this respect. Thus, combination of 4 genes (*entFM*, *nheA*, *hblC*, and *cytK*) was detected in many isolates. This is consistent with findings with a significant number of isolates carrying diarrhoeal toxin-encoding genes [6, 7, 37, 38].

## 5. Conclusion

The contamination of spices and herbs with the food-borne pathogen *Bacillus cereus* is increasingly being reported. The present study revealed that although the percent of prevalence of *B. cereus* in spices is significant, the spices meet food safety recommendations. A high number of multidrug resistant strains carrying diarrhoeal toxin-encoding genes were found. The presence of potentially virulent and antibiotic-resistant*B. cereus* in spices intended for human consumption represents a serious health hazard. The high prevalence of *B. cereus* in various foodstuffs, including spices, can be explained by the origin of *B. cereus*, the soil, and the nonobservance of farm-to-table hygiene practices. It is therefore still possible to prevent food contamination with this organism by applying hygienic principles and use pasteurization technology that not only ensure microbiological safety, but also maintain quality of processed spices.

## Figures and Tables

**Table 1:** Primers used to detect virulence factors.

*Gene*	Sequence	Product length
*cytK*	TGCTAGTAGTGCTGTAACTC	881
	CGTTGTTTCCAACCCAGT	

*nheA*	GGAGGGGCAAACAGAAGTGAA	750
	CGAAGAGCTGCTTCTCTCGT	

*ces*	TTCCGCTCTCAATAAATGGG	634
	TCACAGCACATTCCAAATGC	

*cer*	GCGTACCAAATCACCCGTTC	546
	TGCAGGTGGCACACTTGTTA	

*hblC*	CGCAACGACAAATCAATGAA	421
	ATTGCTTCACGAGCTGCTTT	

*entFM*	AGGCCCAGCTACATACAACG	327
	CCACTGCAGTCAAAACCAGC	

**Table 2:** Presence of *B. cereus* in spice samples (*n* = 200).

Sample type (no)	No. of positive samples (%)	Min log10 CFU/g ± SD^a^	Max log10 CFU/g ± SD^a^
Sumac (*n* = 25)	4 (16%)	2.14 ± 0.1	3.7 ± 0.1
Curry powder (*n* = 25)	14 (56%)	2.18 ± 0.12	3.88 ± 0.05
Cumin (*n* = 25)	6 (24%)	3.15 ± 0.05	4.38 ± 0.15
Turmeric (*n* = 25)	16 (64%)	2.16 ± 0.16	3.52 ± 0.11
Cinnamon (*n* = 25)	12 (48%)	1.2 ± 0.14	3.71 ± 0.05
White pepper (*n* = 25)	6 (24%)	1.42 ± 0.07	4.21 ± 0.16
Chilli (*n* = 25)	8 (32%)	1.7 ± 0.17	3.54 ± 0.09
Black pepper (*n* = 25)	18 (72%)	1.1 ± 0.15	3.8 ± 0.08
Total (*n* = 200)	84/200 (42%)		

SD^a^ e standard deviations.

**Table 3:** Bacterial count and determination of enterotoxin producing *B. cereus*.

Sample	No. of *B. cereus* isolates	Average of *B. cereus*count (log10 CFU/g)	Number of toxin producing *B. cereus*	
			NHE	HBL
Sumac	4	2.93	1	1
Curry powder	25	3.20	9	6
Cumin	6	3.82	3	2
Turmeric	16	2.92	7	4
Cinnamon	12	2.39	6	3
White pepper	6	2.87	3	1
Chilli	8	2.62	4	3
Black pepper	18	2.52	10	3

Note: NHE: nonhaemolytic enterotoxin, HBL: haemolysin BL, +positive, −negative.

**Table 4:** Antibiotic resistance pattern of *B. cereus* isolates.

Antibiotics	No. of resistant isolates (%)
Tetracycline (30 *μ*g)	15 (17.86%)
Penicillin G (10 U)	69 (82.14%)
Vancomycin (30 *μ*g)	17 (20.24%)
Gentamicin	19 (22.62%)
Cefixime	8 (9.52%)
Ciprofloxacin	23 (27.38%)
Cefazolin	14 (16.66%)
Erythromycin (15 *μ*g)	13 (15.47%)
Ampicillin (10 *μ*g)	70 (83.33%)
Chloramphenicol (30 *μ*g)	2 (2.38%)
Nalidixic acid	4 (4.76%)
Imipenem	3 (3.57%)
Enrofloxacin	6 (7.14%)
Oxacillin	10 (11.90%)
Trimethoprim/Sulfamethoxazole	7 (8.33%)
Oleondamycin (15 *μ*g)	2 (2.38%)
Polymixin B (300 U)	1 (1.19%)
Cephalothin (30 *μ*g)	1 (1.19%)
Kanamycin (30 *μ*g)	2 (2.38%)
Streptomycin (10 *μ*g)	3 (3.57%)
Neomycin (30 *μ*g)	2 (2.38%)

**Table 5:** Resistance profile of *B. cereus* strains.

Antibiotics	No. of multiple resistant isolates (%)
P, PB	1 (1.19%)
P, AMP	2 (2.38%)
P, AMP, Te	7 (8.33%)
P, AMP, E, Te	5 (5.95%)
P, AMP, Te, S, E	6 (7.14%)
P, AMP, E, N, S	1 (1.19%)
P, AMP, Te, S, E, K	2 (2.38%)
P, AMP, Te, C, E, N, Va, N	8 (9.52%)

Note: Ol, oleandomycin; Te, tetracycline; Pb, polymixin B; C, chloramphenicol; E, erythromycin; P, penicillin G; Kf, cephalothin; Amp, ampicillin; K, kanamycin; Va, vancomycin; S, streptomycin; N, neomycin.

**Table 6:** Detection of some genes related to enterotoxin production.

Combination of genes	0–4^a^	8–64^a^	>128^a^
*cytK* + *nheA* + *hblC* + *entFM*	6/23 (26%)	3/23 (13%)	14/23 (61%)
*nheA* + *hblC* + *entFM*	4/18 (19%)	5/18 (27%)	9/18 (54%)
*nheA* + *cytK* + *entFM*	1/3 (33%)	ND	2/3 (67%)
*cytK* + *entFM*	ND	1/1 (100%)	ND
*nheA* + *entFM*	5/9 (57%)	1/9 (10%)	3/9 (33%)
*entFM*	9/9 (100%)	ND	ND
*nheA*	35/70 (50%)	ND	35/70 (50%)

Note: ND^b^ -No gene combination detected. ^a^For the Oxoid test, the indexes from 0 to 128 corresponds to the last dilution (among 1/2 serial dilutions) for which the enterotoxin remained present.

## Data Availability

All data generated or analysed during this study are included in this article.
